# Isolation of Three Novel Senecavirus A Strains and Recombination Analysis Among Senecaviruses in China

**DOI:** 10.3389/fvets.2020.00002

**Published:** 2020-01-22

**Authors:** Zhenhua Guo, Xin-xin Chen, Haiyu Ruan, Songlin Qiao, Ruiguang Deng, Gaiping Zhang

**Affiliations:** ^1^Key Laboratory of Animal Immunology of the Ministry of Agriculture, Henan Provincial Key Laboratory of Animal Immunology, Henan Academy of Agricultural Sciences, Zhengzhou, China; ^2^College of Animal Science and Veterinary Medicine, Henan Agricultural University, Zhengzhou, China; ^3^Jiangsu Co-innovation Center for Prevention and Control of Important Animal Infectious Diseases and Zoonoses, Yangzhou, China

**Keywords:** Senecavirus A, emerging disease, vesicular disease, genetic diversity, recombinant

## Abstract

Senecavirus A (SVA), an emerging swine picornavirus of swine, is one of the causative agents of vesicular disease which is clinically indistinguishable from foot-and-mouth disease in pigs. Here, 3 cases of vesicular disease were reported which was caused by SVA in November 2018 in Henan, China. Three new SVA strains were identified and conducted a genetically evolutionary analysis. The isolates shared 98.1–99.0% genomic pairwise identity to each other and had the highest similarity, of 98.3–98.7%, with the American strain KS15-01, respectively. Phylogenetic analysis indicated that the Chinese prevalent strains could be clearly divided into cluster 1, cluster 2, and cluster 3. Furthermore, one isolate (HeNNY-1/2018) and two previously reported strains (HB-CH-2016 and SVA/CHN/10/2017) were identified as recombinants using several algorithms. It revealed that the recombination among SVA strains has occurred in China since 2016 or earlier. The findings of studies updated the prevalent status of SVA in China. Besides, the genetic evolution and recombinant events of SVA should be attracted more attentions in the future.

## Introduction

Senecavirus A (SVA), also known as Seneca Valley virus (SVV), is the only member of the genus *Senecavirus* in the family *Picornaviridae* (1). SVA is a non-enveloped, single-strand and positive-sense RNA virus. The genome size is about 7.3 kb consisting of a single open reading frame (ORF) encoding a polyprotein that is flanked by 5′ and 3′ untranslated regions (UTRs). The polyprotein is subsequently cleaved in a typical picornavirus L4-3-4 layout, namely Leader (Lpro)-P1 region (VP1 to Vp4)-P2 region (2A to 2B)-and P3 region (3A to 3D) (2).

SVA was first isolated as a contaminant of the PER.C6 cell line in 2002 and infrequently associated with porcine vesicular disease (1). However, beginning in late 2014, multiple cases of porcine vesicular disease were reported in Brazil and the America in which SVA was detected in those samples (3–5). Then, SVA is considered to be one of the causative agents of vesicular disease in pigs (5–9). The vesicular disease caused by SVA is clinically indistinguishable from foot-and-mouth disease virus (FMDV), vesicular stomatitis virus (VSV), and swine vesicular disease virus (SVDV) (2, 8). Currently, this virus has been reported in Canada, China, Colombia, Thailand, Viet Nam, and elsewhere, suggesting that SVA-induced disease has already become a worldwide problem (7, 10–12).

In China, the vesicular disease caused by SVA was first reported in Guangdong province in 2015 (12). Since then, increasing cases of SVA infection have been reported in other provinces, including Heilongjiang, Hubei, Henan, Fujian, Hebei, and Anhui etc. (13–18). However, the genomic information is still very limited in these regions except Guangdong province which account for over 70% of Chinese isolates (19). Here, we report 3 apparently unrelated cases of vesicular disease in November 2018 in Henan province, China. Three novel SVA strains were genetically characterized and phylogenetically analyzed. Further, one of the isolates and two strains reported before were all identified as recombinants with unique recombination patterns.

## Materials and Methods

In November 2018, typical vesicular disease outbreaks were reported on three apparently unrelated pig farms (Farm A, B, and C) in Henan province, China in spite of the fact that all pigs had been previously compulsorily vaccinated 2 or 3 times with commercial FMDV vaccine. The geographical distribution of farms and the details of swine herds status were showed in Table 1. Diseased pigs exhibited similar clinical symptoms including lameness, vesicles, and ulcerative lesions on hooves and snouts. The outbreak on farm A was observed in gilts with >125 kg body weight. Pigs in farm B and farm C are commercial pigs with a body weight about 110–120 kg which were ready to market. Morbidity was 20.0% on farm A, 45.6% on farm B, and 18.8% on farm C, with no mortality observed on any farm (Table 1). The infected pigs took about 10 days to recover. The vesicular lesion swabs, vesicular fluids or tissues were sampled to differential diagnosis using specific primers for detection of SVA, FMDV, VSV, and SVDV (15). For virus isolation, the vesicular fluid was diluted with sterile phosphate-buffered saline (PBS) and clarified at 12,000 rpm for 2 min. The supernatant was filtrated by 0.45 μm filters and then incubated with the PK-15 cells. Typical cytopathic effects (CPE) could be observed after 2 or 3 blind passages. Furthermore, the immunofluorescence assay (IFA) was performed with porcine SVA positive serum which was described previously (a kind gift from Dr. Haixue Zheng) (20). The 5th passaged virus was used to do the plaque assay and one-step growth curve as described previously (15, 20). Genome sequences were further determined using primers reported before (15, 21).

**Table 1:** The geographical distribution of 3 pig farms and the detailed status of swine herds.

**Farm**	**Strain name**	**GenBank no**.	**Location**	**Herds size**	**Diseased pigs**	**Body weight**	**Morbidity (infected/total)**	**Samples**
A	HeNNY-1/2018	MK357116	Nanyang, Southwest of Henan	600 sows	Gilts	~125 kg	20.0% (12/60)	Vesicular fluids
B	HeNZMD-1/2018	MK357115	Zhumadian, South of Henan	2,000 finishing	Finishing pigs	~110–120 kg	45.6% (912/2,000)	Vesicular lesion swabs
C	HeNKF-1/2018	MK357117	Kaifeng, East of Henan	600 finishing	Finishing pigs	~110–120 kg	18.8% (113/600)	Vesicular fluids and tissues

Nucleotides sequence alignments for three isolates and other 73 strains (up to December in 2018) available from GenBank were performed using the Multiple Alignment using Fast Fourier Transform (MAFFT) program [Table S1; (22)]. The DNAstar package (DNASTAR, Inc., Madison, WI, USA) was used to conduct homology analysis. The phylogenetic tree was constructed by MEGA 6.0 software using neighbor-joining method and the Kimura-2-parameter nucleotide substitution model with 1,000 bootstrap replicates (23). For recombinant analyses, multiple genome alignment was submitted to screen the potential recombination events by Recombination Detection Program 4 (RDP4). Seven different methods, including RDP, GENECONV, BootScan, Maxchi, Chimera, Siscan, and 3Seq, were employed (24). The recombinant strains were further confirmed by SimPlot 3.5.1 version (25).

## Results and Discussion

**Figure 1:**
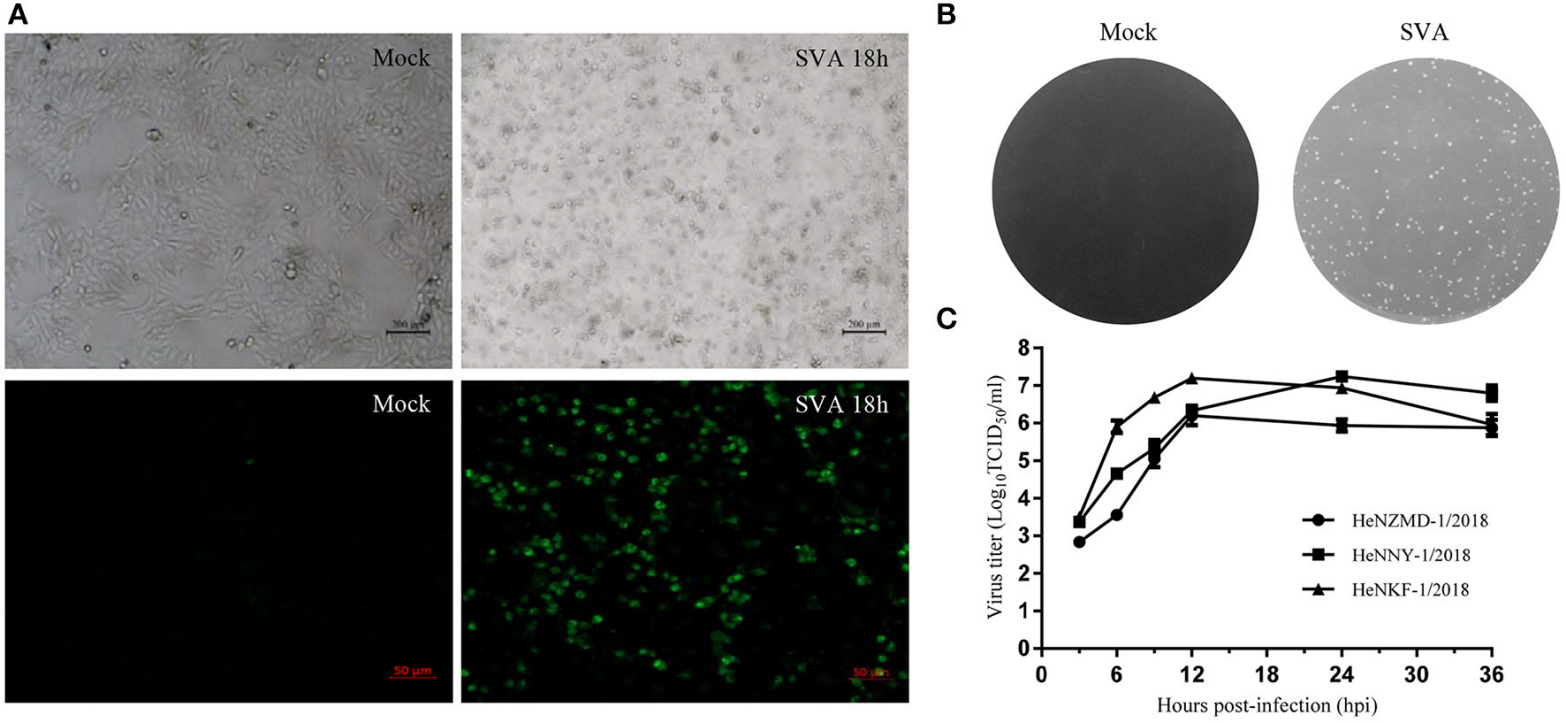
Isolation of SVA strains. **(A)** Representative images of cytopathic effects and immunofluorescence assay. PK-15 cells infected with SVA HeNNY-1/2018 strain at 18 h post-infection. Cells were stained with primary antibody of porcine SVA positive serum. **(B)** Representative plaque morphology in PK-15 cells infected by HeNNY-1/2018 strain at 72 h post-infection. **(C)** One-step growth curve of three SVA isolates on PK-15 cells.

SVA was diagnosed as the causative agent and FMDV, VSV, and SVDV were ruled out by RT-PCR tests. Viral isolation was performed after propagation in PK-15 cells. Three representative SVA strains were isolated and designated as HeNZMD-1/2018, HeNNY-1/2018, and HeNKF-1/2018 (GenBank no. MK357115, MK357116, and MK357117). Typical cytopathic effects (CPE), IFA with specific porcine positive serum against SVA and obvious plaques were observed after infection with SVA at indicated time points (Figures 1A,B and Figure S1). One-step growth curves were obtained after virus infection of PK-15 cells at a multiplicity of infection (MOI) of 0.1. The infected cells were collected at 3, 6, 9, 12, 24, and 36 h post-infection (hpi). The viral loads were titrated by 50% tissue culture infective dose (TCID_50_) assay with maximum viral titers obtained that were about 10^6.21^ TCID_50_/ml (HeNZMD-1/2018), 10^7.24^ TCID_50_/ml (HeNNY-1/2018), and 10^7.20^ TCID_50_/ml (HeNKF-1/2018), respectively (Figure 1C).

The genome size of these isolates is 7,285 nucleotides (nt) consisting of a long 5′ UTR of 668 nt, an ORF encoding a 2,181 amino acid polyprotein and a short 3′ UTR of 71 nt, which exhibited similar genome organization to other SVA strains. Genome sequence alignment showed that the isolates shared 98.1–99.0% nucleotide identity to each other, but diverged by 3.6–3.9% from the first reported strain CH-01-2015 (GenBank no: KT321458) in China and by 6.4–6.6% from the prototype strain SVV-001 (GenBank no: NC_011349). Surprisingly, the 3 isolates showed the highest similarity, of 98.3–98.7%, with the 2015 American strain KS15-01 (GenBank no: KX019804).

**Figure 2:**
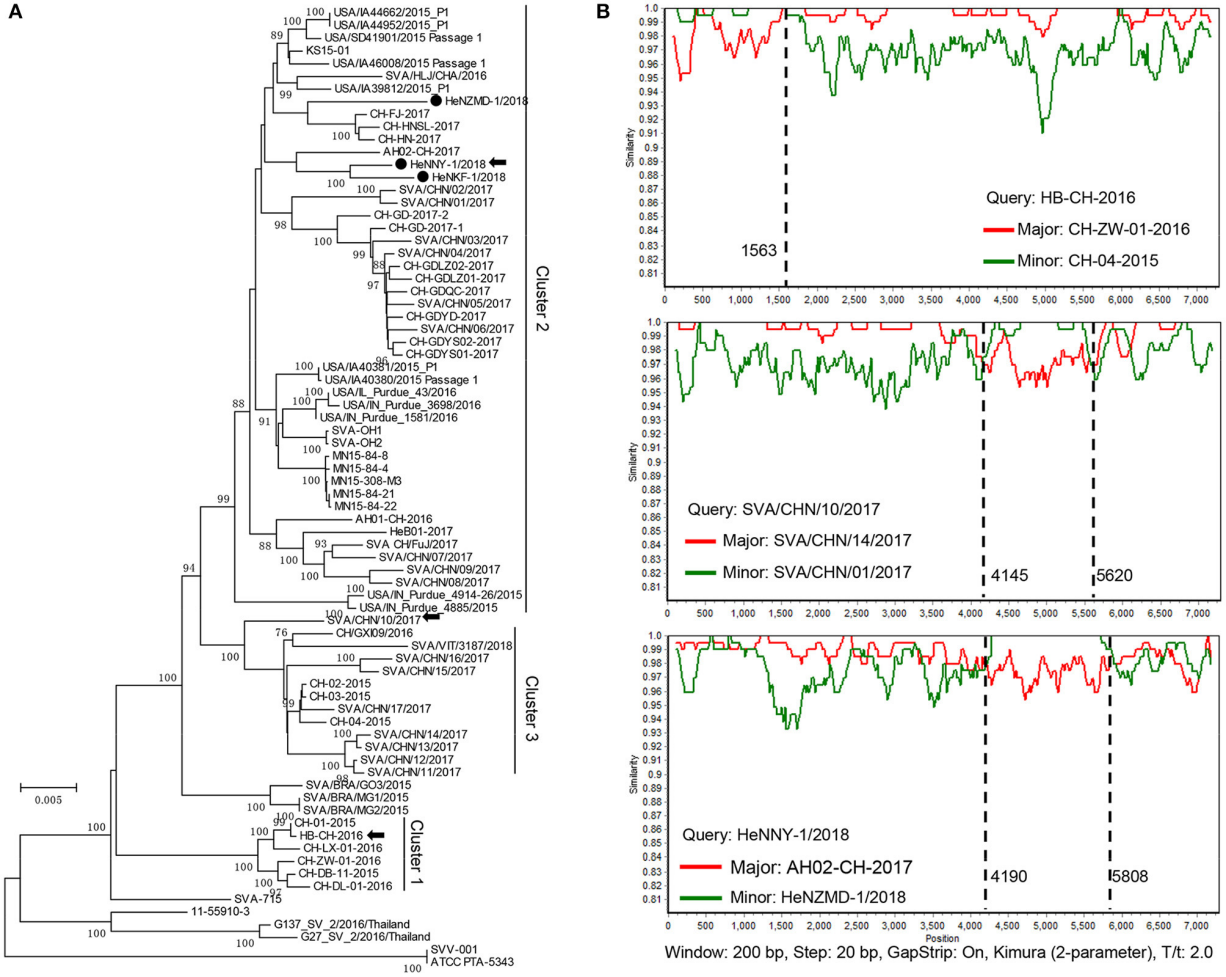
Phylogenetic and recombination analysis of complete SVA genomes. **(A)** Reported SVA strains from China could be divided into 3 clusters. Phylogenetic trees were constructed with MEGA 6.0 software using neighbor-joining method, with 1,000 bootstrap replicates. Newly isolated strains are marked by black solid circles (•). Recombinant strains are indicated with black arrows. **(B)** Recombination analyses of SVA strains in this study. Crossover regions were identified by RDP4 and SimPlot 3.5.1 softwares. The *X-axis* shows the location of the query strain, and the *Y-axis* indicates the percentage of similarity. Reference strain, the major parental strain (red) and the minor parental strain (green).

Sequences of the three isolates described here and other 73 GenBank strains were compared using MEGA6.0 software [Table S1; (23)]. As shown in Figure 2A, the prevalent strains in China could be clearly divided into 3 clusters (clusters 1–3), revealing a high genetic diversity and sequence complexity among SVA strains prevalent in China. The new isolates described here were grouped into cluster 2, are closely related to the strains CH-FJ-2017 and AH02-CH-2017 (GenBank no. KY747510, MF460449) and distant from the early reported strain CH-01-2015, CH-02-2015, and CH-03-2015 (GenBank no. KT321458, KX173339, and KX173338) in China. It is still unknown how the SVA was introduced into China. One hypothesis is the international trading (breeding, feed ingredients, pork, and pork products etc.) or international communication of swine industry practitioners. Further researches and retrospective studies may answer when the virus starts to circulate in pig herds in China.

Genomic recombination is a genetic feature of picornaviruses, which has been reported in enteroviruses, aphthoviruses, parechoviruses, and cardioviruses (26). However, recombination events among Senecaviruses are still poorly understood. Here, three new isolates and another 43 Chinese prevalent strains previously submitted to GenBank (up to December in 2018) were screened by RDP4.0 using several algorithms (24). The statistical results strongly supported that HeNNY-1/2018 (isolated in this study), HB-CH-2016 (GenBank no. KX377924, isolated in Hubei in 2016), and SVA/CHN/10/2017 (GenBank no. MG765559, isolated in China in 2017) are three recombinants exhibiting unique genetic recombination patterns (*P* < 0.001, recombinant score >0.7) (Table S2). Meanwhile, recombination events were further confirmed using SimPlot 3.5.1 software (25). The detailed beginning and ending breakpoints and parental strains were shown in Figure 2B. The minor fragments of HeNNY-1/2018 (region 4,190–5,808 nt) and SVA/CHN/10/2017 (region 4,145–5,620 nt) showed a similar recombination pattern (crossover the P2 and P3 regions of the genome), including the partial 2C, 3A, 3B, and partial 3C genes. While the recombination within the HB-CH-2016 (region 1-1563 nt) mainly occurred within the 5′ of the genomic region containing the complete 5′ UTR, Lpro, and partial P1 region (VP4 and partial VP2 genes). Recently, Wang et al. also described a mosaic strain, HeN-1/2018, that exhibited a recombination region (960-2354 nt) within the P1 genome region containing VP4 (partial), VP2, and VP3 (partial) genes (27). Combined with our studies, the recombination breakpoints were mapped to P1, P2, and P3 regions. However, the lack of SVA sequence data prevents estimating recombination frequencies. More research needs to be done to map the recombination hotspots over the SVA genome.

In China, the first SVA infection was reported in 2015 in Guangdong Province (12). Since then, other cases have been sporadically reported in several regions with a significant increase in numbers and geographical distributions (15, 16, 18). The high density of pig farms and frequent movement of live pigs through different regions will contribute to the SVA spread in China. Moreover, the key role that recombination plays in the microevolution of picornaviruses and emergence of novel variants is of great concern, especially since it sometimes leads to severe pathogenicity (26). Therefore, SVA recombination events should be monitored carefully.

In conclusion, we reported 3 cases of vesicular disease caused by SVA in November 2018 in China. Three new SVA strains were identified and conducted a genetical evolutionary analysis. Our studies demonstrated that high levels of genetic diversity among SVA strains in China. Furthermore, one isolate and two previously reported strains were identified as recombinants with unique recombination patterns. These results suggest that SVA recombination events have been occurring in China since as early as 2016. The frequent recombination incidences will lead to the emergence of novel variants and increase the complexity of SVA transmission, which pose a challenge to the prevention and intervention of SVA infection in future.

## Data Availability Statement

All data generated or analyzed during this study are included in this published article/Supplementary Material.

## Ethics Statement

This animal study was reviewed and approved by the Institutional Animal Care and Use Committee of Henan Academy of Agricultural Sciences. Written informed consent was obtained from the owners for the participation of their animals in this study.

## Author Contributions

ZG performed the experiments. ZG and XC wrote the manuscript. HR, SQ, and RD analyzed the data. GZ designed and supervised the experiments. All authors read and approved the final manuscript.

### Conflict of Interest

The authors declare that the research was conducted in the absence of any commercial or financial relationships that could be construed as a potential conflict of interest.

## Supplementary Material

Figure S1 Representative images of cytopathic effects and immunofluorescence assay. PK-15 cells infected with SVA HeNNY-1/2018, HeZMD-1/2018, and HeNKF-1/2018 strains at 18 h post-infection. Cells were stained with primary antibody of porcine SVA positive serum.

Table S1 Sequence information in this study.

Table S2 The recombination events detected by RDP4.0 after complete genome sequence alignment of SVA strains.
