# Supplementary figures and images for: Isolation of Three Novel Senecavirus A Strains and Recombination Analysis Among Senecaviruses in China
Source: Front Vet Sci. 2020 Jan 22;7:2. doi: 10.3389/fvets.2020.00002 (PMC6996486; doi:10.3389/fvets.2020.00002)

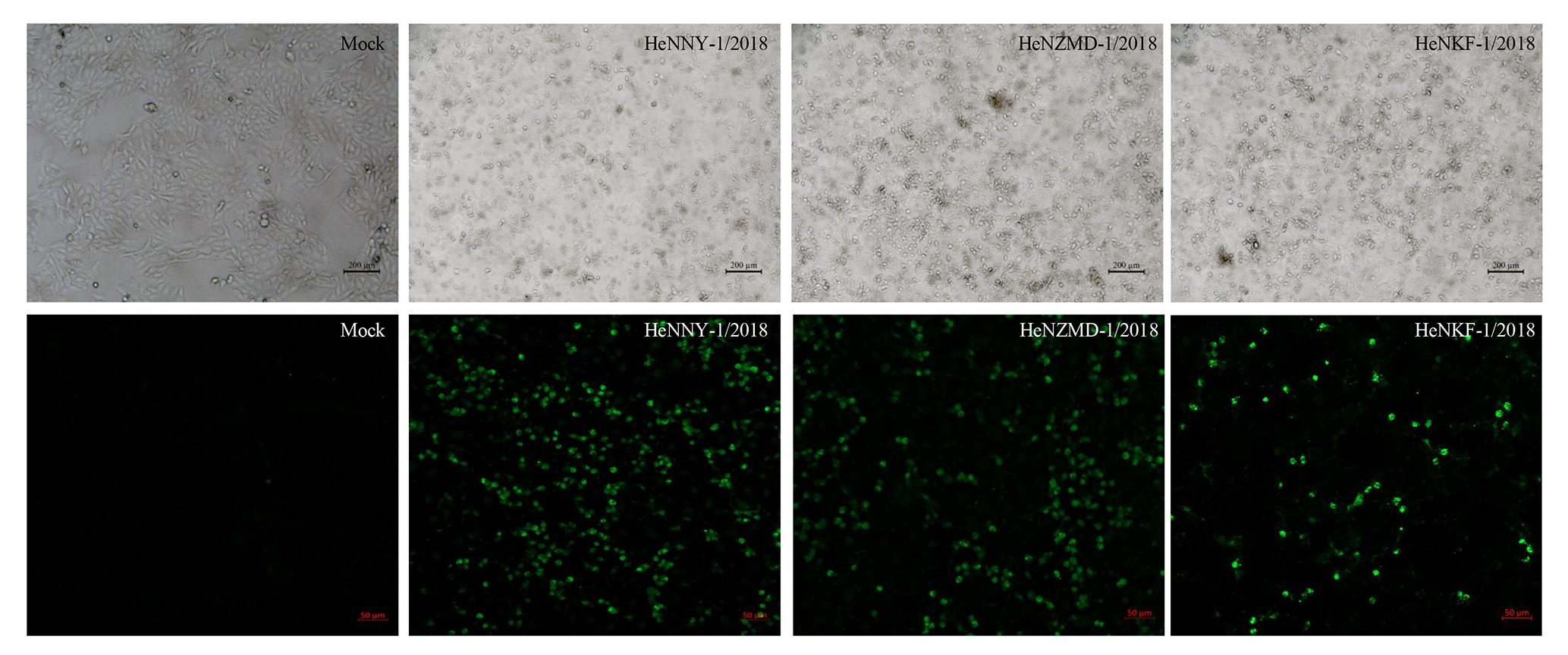

Supplement: Figure S1 — Representative images of cytopathic effects and immunofluorescence assay. PK-15 cells infected with SVA HeNNY-1/2018, HeZMD-1/2018, and HeNKF-1/2018 strains at 18 h post-infection. Cells were stained with primary antibody of porcine SVA positive serum. [file Image_1.TIF]
