# Supplementary material for: Isolation of Three Novel Senecavirus A Strains and Recombination Analysis Among Senecaviruses in China
Source: Front Vet Sci. 2020 Jan 22;7:2. doi: 10.3389/fvets.2020.00002 (PMC6996486; doi:10.3389/fvets.2020.00002)
Supplement: Table S1 — Sequence information in this study. [file Table_1.DOC]

Supplemental Table S1: Sequence information in this study.

| No. | Strain name | Accession No. | Origin | Year |
| --- | --- | --- | --- | --- |
| 1 | SVV-001 | NC_011349 | USA | 2008 |
| 2 | SVA-715 | KY172968 | USA | 2014 |
| 3 | KS15-01 | KX019804 | USA | 2015 |
| 4 | SVA-OH2 | KU058183 | USA | 2015 |
| 5 | SVA-OH1 | KU058182 | USA | 2015 |
| 6 | USA/IN_Purdue_4914-26/2015 | KY618834 | USA | 2015 |
| 7 | USA/IL_Purdue_43/2016 | KY618835 | USA | 2016 |
| 8 | USA/IN_Purdue_1581/2016 | KY618836 | USA | 2016 |
| 9 | USA/IN_Purdue_3698/2016 | KY618837 | USA | 2016 |
| 10 | ATCC PTA-5343 | KU954086 | USA | 2016 |
| 11 | USA/IA39812/2015_P1 | KU954087 | USA | 2015 |
| 12 | USA/IA40381/2015_P1 | KU954088 | USA | 2015 |
| 13 | USA/IA44662/2015_P1 | KU954089 | USA | 2015 |
| 14 | USA/IA44952/2015_P1 | KU954090 | USA | 2015 |
| 15 | USA/IN_Purdue_4885/2015 | KX223836 | USA | 2015 |
| 16 | MN15-84-4 | KU359210 | USA | 2015 |
| 17 | MN15-84-8 | KU359211 | USA | 2015 |
| 18 | MN15-84-21 | KU359212 | USA | 2015 |
| 19 | MN15-84-22 | KU359213 | USA | 2015 |
| 20 | MN15-308-M3 | KU359214 | USA | 2015 |
| 21 | USA/IA40380/2015 Passage 1 | KT757280 | USA | 2015 |
| 22 | USA/SD41901/2015 Passage 1 | KT757281 | USA | 2015 |
| 23 | USA/IA46008/2015 Passage 1 | KT757282 | USA | 2015 |
| 24 | 11-55910-3 | KC667560 | Canada | 2011 |
| 25 | SVA/BRA/GO3/2015 | KR063109 | Brazil | 2015 |
| 26 | SVA/BRA/MG2/2015 | KR063108 | Brazil | 2015 |
| 27 | SVA/BRA/MG1/2015 | KR063107 | Brazil | 2015 |
| 28 | G137_SV_2/2016/Thailand | MF416220 | Thailand | 2016 |
| 29 | G27_SV_2/2016/Thailand | MF416218 | Thailand | 2016 |
| 30 | SVA/VIT/3187/2018 | MH704432 | Viet Nam | 2018 |
| 31 | CH-01-2015 | KT321458 | China,Guangdong | 2015 |
| 32 | SVA/HLJ/CHA/2016 | KY419132 | China,Heilongjiang | 2016 |
| 33 | CH/GXI09/2016 | KY038016 | China | 2016 |
| 34 | HB-CH-2016 | KX377924 | China,Hubei | 2016 |
| 35 | CH-GDYS02-2017 | MG428685 | China,Guangdong | 2017 |
| 36 | CH-GDYS01-2017 | MG428684 | China,Guangdong | 2017 |
| 37 | CH-GDYD-2017 | MG428683 | China,Guangdong | 2017 |
| 38 | CH-GDQC-2017 | MG428682 | China,Guangdong | 2017 |
| 39 | CH-GDLZ02-2017 | MG428681 | China,Guangdong | 2017 |
| 40 | CH-GDLZ01-2017 | MG428680 | China,Guangdong | 2017 |
| 41 | CH-HN-2017 | KY747511 | China,Henan | 2017 |
| 42 | CH-HNSL-2017 | KY747512 | China,Henan | 2017 |
| 43 | CH-FJ-2017 | KY747510 | China,Fujian | 2017 |
| 44 | CH-ZW-01-2016 | KX751946 | China,Guangdong | 2016 |
| 45 | CH-LX-01-2016 | KX751945 | China,Guangdong | 2016 |
| 46 | CH-DL-01-2016 | KX751944 | China,Guangdong | 2016 |
| 47 | CH-DB-11-2015 | KX751943 | China,Guangdong | 2015 |
| 48 | CH-GD-2017-2 | MF189001 | China,Guangdong | 2017 |
| 49 | CH-GD-2017-1 | MF189000 | China,Guangdong | 2017 |
| 50 | SVA CH/FuJ/2017 | MH490944 | China,Fujian | 2017 |
| 51 | SVA/CHN/17/2017 | MG765566 | China,Guangdong | 2017 |
| 52 | SVA/CHN/16/2017 | MG765565 | China,Guangdong | 2017 |
| 53 | SVA/CHN/15/2017 | MG765564 | China,Guangdong | 2017 |
| 54 | SVA/CHN/14/2017 | MG765563 | China,Guangdong | 2017 |
| 55 | SVA/CHN/13/2017 | MG765562 | China,Guangdong | 2017 |
| 56 | SVA/CHN/12/2017 | MG765561 | China,Guangdong | 2017 |
| 57 | SVA/CHN/11/2017 | MG765560 | China,Guangdong | 2017 |
| 58 | SVA/CHN/10/2017 | MG765559 | China,Guangdong | 2017 |
| 59 | SVA/CHN/09/2017 | MG765558 | China,Guangdong | 2017 |
| 60 | SVA/CHN/08/2017 | MG765557 | China,Guangdong | 2017 |
| 61 | SVA/CHN/07/2017 | MG765556 | China,Guangdong | 2017 |
| 62 | SVA/CHN/06/2017 | MG765555 | China,Guangdong | 2017 |
| 63 | SVA/CHN/05/2017 | MG765554 | China,Guangdong | 2017 |
| 64 | SVA/CHN/04/2017 | MG765553 | China,Guangdong | 2017 |
| 65 | SVA/CHN/03/2017 | MG765552 | China,Guangdong | 2017 |
| 66 | SVA/CHN/02/2017 | MG765551 | China,Guangdong | 2017 |
| 67 | SVA/CHN/01/2017 | MG765550 | China,Guangdong | 2017 |
| 68 | HeB01-2017 | MF967574 | China | 2017 |
| 69 | AH02-CH-2017 | MF460449 | China | 2017 |
| 70 | AH01-CH-2016 | MF460448 | China | 2016 |
| 71 | CH-04-2015 | KX173340 | China,Guangdong | 2015 |
| 72 | CH-02-2015 | KX173339 | China,Guangdong | 2015 |
| 73 | CH-03-2015 | KX173338 | China,Guangdong | 2015 |
| 74 | **HeNZMD-1/2018** | **MK357115** | **China,Henan** | **2018** |
| 75 | **HeNNY-1/2018** | **MK357116** | **China,Henan** | **2018** |
| 76 | **HeNKF-1/2018** | **MK357117** | **China,Henan** | **2018** |

The isolated strains were in bold.
