# Supplementary material for: Isolation of Three Novel Senecavirus A Strains and Recombination Analysis Among Senecaviruses in China
Source: Front Vet Sci. 2020 Jan 22;7:2. doi: 10.3389/fvets.2020.00002 (PMC6996486; doi:10.3389/fvets.2020.00002)
Supplement: Table S2 — The recombination events detected by RDP4.0 after complete genome sequence alignment of SVA strains. [file Table_2.DOC]

**Table S2.** The recombination events detected by RDP4.0 after complete genome sequence alignment of SVA strains.

| Recomninant | Major  parent | Minor  parent | Position | Detection methods (Av.P-Val) | | | | | | | Recombiant score |
| --- | --- | --- | --- | --- | --- | --- | --- | --- | --- | --- | --- |
|  |  |  |  | RDP | GENECONV | BootScan | MaxChi | Chimaera | SiScan | 3Seq |  |
| SVA/CHN/10/2017 | SVA/CHN/14/2017 | SVA/CHN/01/2017 | 4145-5620 | 9.54×10^-11^ | 1.99×10^-10^ | NS | 4.23×10^-7^ | 8.75×10^-7^ | 6.74×10^-8^ | 5.33×10^-15^ | 0.74 |
| HB-CH-2016 | CH-ZW-01-2016 | CH-04-2015 | 1-1563 | 9.54×10^-11^ | 4.41×10^-10^ | 5.14×10^-13^ | 4.23×10^-7^ | 8.75×10^-7^ | 6.92×10^-8^ | 5.33×10^-15^ | 0.74 |
| HeNNY-1/2018 | AH02-CH-2017 | HeNZMD-1/2018 | 4190-5808 | 1.05×10^-6^ | 2.82×10^-10^ | NS | 1.97×10^-6^ | 3.14×10^-6^ | 9.53×10^-10^ | 7.41×10^-10^ | 0.75 |

NS：not significant
